# Shifts in Structure and Assembly Processes of Root Endophytic Community Caused by Climate Warming and Precipitation Increase in Alpine Grassland

**DOI:** 10.3390/microorganisms12091780

**Published:** 2024-08-28

**Authors:** Xiaoting Wei, Bing Han, Jinxin Zhang, Xinqing Shao

**Affiliations:** 1 Institute of Ecological Conservation and Restoration, Chinese Academy of Forestry, Beijing 100091, China; weixiaoting6@163.com; 2 College of Grassland Science and Technology, China Agricultural University, Beijing 100193, China; hbing_edu@163.com

**Keywords:** alpine grassland, endophytic community, assembly processes, climate warming, precipitation increase

## Abstract

Climate change poses great challenges to the survival of plants. Plant endophytes play important roles in improving plant adaptability. However, our knowledge of the effects of climate change on endophytic community structures is limited. Relying on a field experimental platform simulating climate warming, precipitation increases, and their combination in an alpine grassland, the root endophytic bacterial community structures and assembly processes of three coexisting plant species (*Elymus nutans*, *Kobresia humilis*, and *Melissilus ruthenicus*) were measured. The results indicated that Proteobacteria was the dominant phylum, with a relative abundance ranging from 50% to 80%, followed by Actinobacteria and Bacteroidetes. Bacterial diversity decreased significantly under the combined treatment for all three plant species, with the largest reduction observed in *E. nutans*. The climate manipulation treatments had a minimal effect on the endophytic bacterial community structures. The relative abundance of Burkholderiaceae increased significantly under the combined treatment for the three plant species. Moreover, the endophytic community assembly processes changed from stochastic dominated under control plots to deterministic dominated under the combined plots for *E. nutans*, while this shift was reversed for *M. ruthenicus*. The root endophytic bacterial community was affected by the soil’s available nitrogen and stoichiometric ratio. These results revealed that the sensitivity of endophyte community structures to climate change varies with host plant species, which has implications for plant fitness differences.

## 1. Introduction

Plant endophytes are the symbiotic microbiota that inhabit plant tissues, including intercellular apoplasts and xylem vessels, without causing disease symptoms [1]. Symbiotic microorganisms are crucial for the survival, evolution, and reproduction of their host plants and are considered the “second genome” of the plant [2,3]. At the individual plant level, microorganisms colonizing plant roots improve host fitness by promoting nutrient absorption, producing phytohormones, and regulating adaptive plant traits [4,5]. For example, root endophytes control root branching by regulating the induction of the phytohormone ethylene, which is important for plant adaptation to abiotic stress and environmental changes [6]. In addition, root microbes can regulate the stress response to low light and the growth compensation of aboveground plant organs through long-distance signaling along the microbiome–root–shoot axis at the expense of some defensive functions [7]. At the plant community level, plant endophytes can drive the plant community structure by shifting the relative fitness and niche of coexisting plant species [8,9,10].

An increasing number of studies have investigating the factors and underlying mechanisms influencing the diversity, composition, and structure of endophytic communities and have found that bacterial endophytic communities are structured according to host species [11,12], tissue type [13], and environmental factors [14]. The secondary metabolites of plants include coumarins, glucosinolates, benzoxazines, snake toxins, and triterpenoids, which are key regulators of endophytic communities [15]. Root dry matter content and root C/N ratio have also been reported to be primary predictors of root microbial community composition [16]. Interspecies trophic interactions, such as cross-feeding or competition for niche space, affect bacterial co-occurrence and community structure [17]. Community assembly processes based on niche and neutral theories have been used to explain the changes in microbial communities. Niche theory emphasizes that deterministic processes, such as environmental filtering, determine the construction of microbial communities [18], while neutral theory posits that microbial community dynamics are mainly controlled by stochastic processes, such as dispersal and gene drift [19]. The consensus is that stochastic and deterministic processes drive microbial communities together; however, their relative influence is context-dependent [20].

Temperature and precipitation are important climatic factors affecting endophytic communities. Changes in temperature and precipitation patterns can directly change the endophytic community structure owing to the sensitivities of the various microorganisms or indirectly change it by affecting plant hormones and exudation [21]. For example, the production of salicylic acid decreases under a warming climate, which is involved in endophytic microbiome assembly [22]. The endophyte diversity of dominant plant species in the Inner Mongolian steppes negatively correlated with mean annual precipitation [23]. Anthropogenic global climate change is accelerating, with an expected rise in the average global temperature of 1.5–2 °C by the end of the century [24]. The frequency of drought and extreme precipitation events has increased because of global warming, which hinder plant growth and development. Plant responses to climate change can be reflected in morphology, physiological characteristics, phenology, and reproduction. In addition, the regulation of endophytic community composition is an important adaptation strategy for plants to cope with climate change [25]. Drought causes significant enrichment of Actinobacteria, which reportedly increase plant drought resistance [26,27]. Pezicula and Paracoccus were enriched in the root of Rhododendron to resist heat stress [28]. A study reporting the response of root endophytic fungi to experimental warming of common grasses in the Rocky Mountains of the United States suggested that there were no significant changes in fungal composition or diversity due to experimental warming [29]. In fact, the response of the endophytic community to climate change is plant-specific [30,31]. This differential response might affect plant coexistence and community succession under climate change.

Located in southwest China, the Qinghai-Tibet Plateau is characterized by high altitudes and low temperatures. Climate warming is more pronounced in this area, and temperature has increased at a rate of 0.4 °C per decade, more than twice the global average rate [32,33]. The Tibetan Plateau is considered a hot spot for climate change studies due to its climate sensitivity. According to a model prediction, the climate of the Qinghai-Tibet Plateau is projected to be even warmer and moister in the future [34]. Previous studies have suggested that different plant functional groups respond differently to temperature and precipitation. For example, grass abundance increases while sedge and forb abundance decrease under a warm and dry climate [35]. Therefore, we targeted representative plant species of grasses (*Elymus nutans*), sedge (*Kobresia humilis*), and leguminous (*Melissilus ruthenicus*) in natural alpine grassland to explore the responses of the root endophytic communities of the three plant species to climate change. We attempted to find some explanations of plant adaptation differences from the perspective of endophytic bacteria. Based on a controlled field experiment of simulated climate warming and precipitation increases in alpine grassland, the most important ecosystem on the Qinghai-Tibet Plateau, this study measured the diversity and composition of the three plant species using 16S rRNA sequencing. We aimed to elucidate the (1) responses of root endophytic bacterial community composition of coexisting plant species to climate change; (2) assembly processes of root endophytic bacterial communities; (3) factors driving the root endophytic bacterial communities’ structure and composition.

## 2. Materials and Methods

### 2.1. Study Site

**Figure 1:**
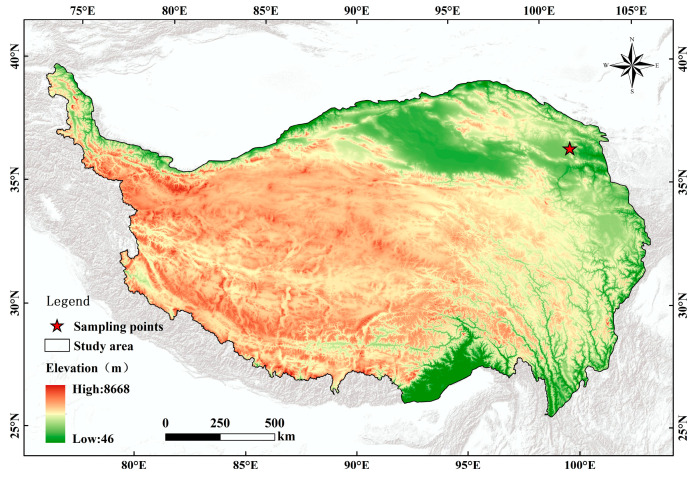
Geographical location of the Qinghai-Tibet Plateau and the experimental site of this study.

This study was conducted on an experimental field platform in an alpine meadow in Qinghai province, China (36°55′ N, 100°57′ E; 3029 m above sea level) (Figure 1). The area had a plateau continental climate characterized by a dry and windy spring, cool summer, short autumn, and long winter. The mean annual temperature was 1.4 °C, and the annual mean precipitation was 410 mm. The highest average temperature generally occurred in July (27 °C) and the lowest in January (−29 °C). Rainfall and heat occur during the same period, with 80% of the precipitation occurring between June and September. Common plant species on the field platform include *Poa crymophila* Keng, *Elymus nutans*, *Leymus secalinus*, *Stipa purpurea*, *Potentilla chinensis*, *Melissilus ruthenicus*, *Astragalus polycladus*, *Kobresia humilis*, and *Gentiana squarrosa*. The soil is classified as a Mat-Gryic Cambisol, with average pH and total carbon, nitrogen, and phosphorus concentrations of 7.7, 40 g·kg^−1^, 3.5 g·kg^−1^, and 0.23 g·kg^−1^, respectively.

### 2.2. Experimental Design and Sampling

In 2014, 16 round plots with a diameter of 2.2 m were arranged in a randomized block design in a 30 m × 30 m alpine meadow. The study area was fenced in 2012 to prevent livestock disturbances. There were four treatments: ambient temperature and precipitation (CK), individual warming (W), individual precipitation increase (P), and a combination of warming and precipitation increase (WP). Four replicate plots were used for each treatment. Open-top chambers (OTCs) (bottom diameter, 2.2 m; top diameter, 1.5 m; height, 0.7 m) were permanently installed in the W and WP plots. Water (25 × 10^3^ cm^3^) was added to the P and WP plots once weekly from June to August, accounting for approximately 20% of annual rainfall.

In August 2021 (growing season), the roots of *E. nutans*, *K. humilis*, and *M. ruthenicus* were dug up from a depth of 15 cm and stored in an incubator on dry ice. Root samples were then transported to the laboratory for surface sterilization using the following procedure: flushing with running water, 75% ethanol for 1 min, 3.25% sodium hypochlorite for 3 min, 75% ethanol for 30 s, and sterile water for 1 min. The sterilized roots were stored in a freezer at −80 °C until DNA extraction of root endophytes. Soil samples from a 15 cm depth were air-dried for property measurement. Soil water content (SWC) was measured by gravimetry. A glass electrode (Orion Star A215, Thermo-Fisher Scientific, Waltham, MA, USA) was used for soil pH determination in deionized water at a 1:5 (*wt*/*vol*) ratio. Soil total carbon (TC) and nitrogen (TN) contents were measured with an elemental auto-analyzer (Vario MAXCN; Elementar, Langenselbold, Germany). An AA3 flow injection analyzer (Flowsys, Ecotech, Germany) was used to determine soil NH_4_^+^-N and NO_3_^−^-N contents. The soil total phosphorus (TP) content was measured using HClO_4_-H_2_SO_4_ colorimetry, while the soil available phosphorus (AP) was measured using Mo-Sb colorimetry after extraction with 0.5 mol/L NaHCO_3_.

### 2.3. DNA Extraction and Gene Sequencing

Genomic DNA was extracted from the root endophytes of plants using an OMEGA Soil DNA Kit D5625-01 (Omega Bio-Tek, Norcross, GA, USA). The targeting primer set 799F (5′-AACMGGATTAGATACCCKG-3′) and 1193R (5′-ACGTCATCCCCACCTTCC-3′) was used to amplify the V5-V7 fragment of the 16S rRNA gene. The PCR amplicons were purified and sequenced using the Illumina MiSeq platform in a paired-end 2 × 250 bp sequencing format. QIIME2 (2019.4) (https://docs.qiime2.org/2019.4/tutorials/) (10 October 2021) was used for the sequence processing. Raw sequence filtering, denoising, merging, and chimera removal were performed using the DADA2 plugin (https://github.com/benjjneb/dada2) (10 October 2021). Taxonomic annotation was performed for the generated nonsingleton amplicon sequence variants (ASVs) using the SILVA Release 132 database.

### 2.4. Data Statistical Analyses

The Shannon–Wiener index and Bray–Curtis dissimilarity of the bacterial communities were estimated using the diversity plugin in QIIME2 (2019.4). Differences in bacterial community composition among treatments were visualized using principal coordinate analysis (PCoA) plots based on the Bray–Curtis distance. Significance tests were conducted using permutational multivariate analysis of variance (PERMANOVA) in the “vegan” package in R (4.2.2). PERMANOVA evaluates the explanation of different grouping factors of sample differences by calculating the distance matrix or similarity matrix between samples, and it uses the permutation test to calculate the significance *p*-value. A *p* value of less than 0.05 indicates significant differences between groups. The normalized stochasticity ratio (NST) was calculated to quantify the relative importance of stochastic processes in governing the bacterial community assembly [36]. Differences in the relative abundance of the top ten families among the four treatments were compared using Dunn’s and Kruskal–Wallis tests. To identify the specialist bacterial species of the three plant species, we calculated the specificity (Equation (1)) and occupancy (Equation (2)) of the ASVs and drew SPEC-OCCU diagrams. ASVs with both specificity and occupancy > 0.7 were identified as specialist species [37].

(1) $$Specificity=\frac{{Nindividuals}_{S,\;H}}{{Nindividuals}_{S}}$$

(2) $$Occupacy=\frac{{Nsites}_{S,\;H}}{{Nsites}_{H}}$$

where Nindividuals_S, H_ indicates the average abundance of ASV species (S) in all samples of plant H, and Nindividuals_S_ indicates the sum of the average abundance of individual S over all three plant species. Nsites_S, H_ indicates the number of samples where S is present in plant H, and Nsites_H_ indicates the total number of samples from plant H.

Significance tests were conducted to analyze the effects of warming, increased precipitation, and their combination on bacterial community diversity. ANOVA and Duncan’s multiple comparison tests were performed using SPSS (IBM SPSS Statistics 20, Chicago, IL, USA) at a significance level of 0.05. Spearman’s rank correlation analysis explored the relationship between bacterial alpha diversity and edaphic factors. The Mantel test was conducted to reveal the correlations between edaphic factors and bacterial community composition using the “vegan” package in R (4.2.2). A *p*-value less than 0.05 indicated that the difference was significant. Plots were generated using GraphPad Prism 8 and Adobe Illustrator software (2020).

## 3. Results

### 3.1. Diversity and Composition of Root Endophytic Bacterial Community

**Figure 2:**
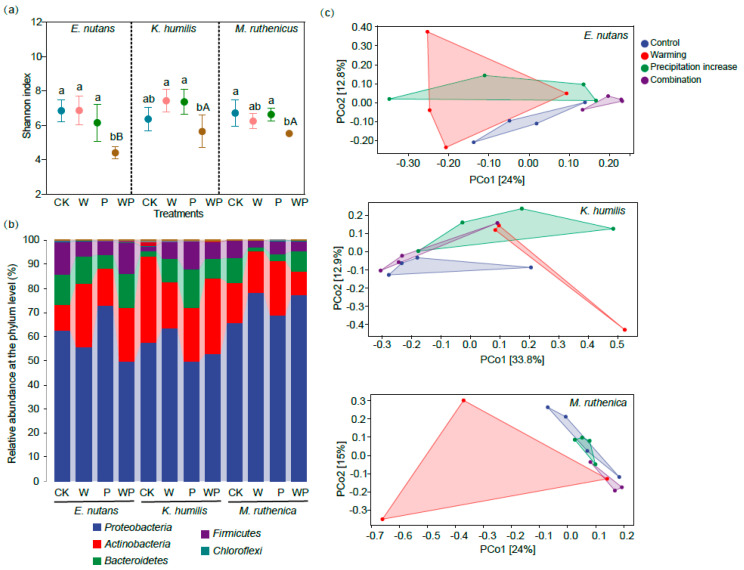
Endophyte Shannon diversity (**a**), taxonomic composition at the phylum level (**b**), and principal coordinate analysis (PCoA) plots of community composition based on the Bray–Curtis distance (**c**) across the treatments. Different lowercase letters indicate significant differences between groups (*p* < 0.05). Different uppercase letters indicate significant difference between plant species (*p* < 0.05). CK, control; W, warming; P, precipitation increase; WP, a combination of warming and precipitation increase.

**Figure 3:**
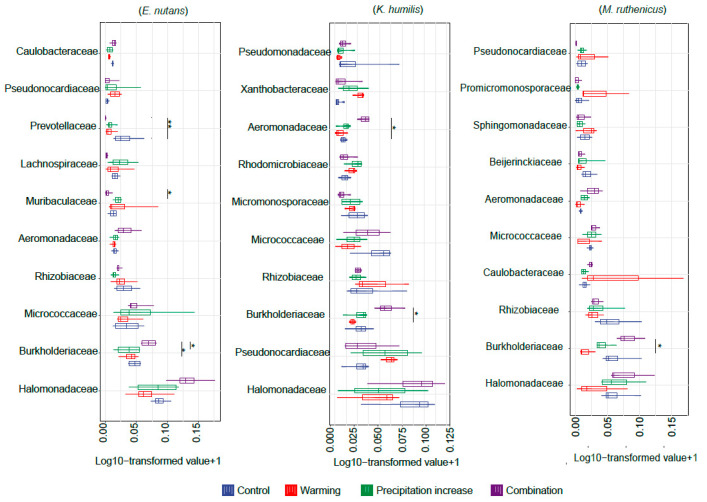
Differences in the relative abundance of bacterial families between treatments for each plant species according to the Duncan multiple comparison tests. Significant differences at the * 0.05 and ** 0.01 levels.

The Shannon index of the bacterial communities was significantly lower for *E. nutans* (−35.57%) and *M. ruthenicus* (−17.74%), while a slight yet insignificant decrease was observed for *K. humilis* under the combined treatment (Figure 2a). Proteobacteria was the dominant phylum, with a relative abundance ranging from 50% to 80%, followed by Actinobacteria, Bacteroidetes, and Firmicutes (Figure 2b). The PCoA plots and results of the PERMANOVA analysis suggested that individual warming and increased precipitation showed no significant effects on the overall bacterial community composition of the three plant species (Table 1). In contrast, the combined treatment changed the bacterial community composition of *E. nutans* (Figure 2c). In addition, individual warming and precipitation increase treatments had little effect on the relative abundance of the dominant taxa, which is a different result from that of the combined treatment. The relative abundance of Burkholderiaceae was significantly higher under the combined treatment for all the three plant species. The relative abundances of Prevotellaceae and Muribaculaceae decreased for *E. nutans*, and the relative abundance of Aeromonadaceae increased for *K. humilis* (Figure 3).

### 3.2. Assembly Processes of Root Endophytic Bacterial Community

**Figure 4:**
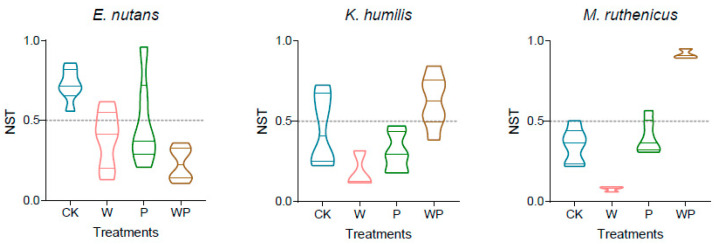
Normalized stochasticity ratio (NST) of endophytic bacterial communities under different treatments for each plant species. CK, control; W, warming; P, precipitation increase; WP, a combination of warming and precipitation increase.

The assembly processes of the root endophytic bacterial communities were altered by climate change (Figure 4). For *E. nutans*, the average NST values were 0.77, 0.37, 0.53, and 0.24 for the CK, W, P, and WP treatment, respectively, implying that community assembly processes shifted from stochastic domination to deterministic domination under the individual warming and combined treatments. For *K. humilis*, the average NST values in the CK, W, P, and WP treatments were 0.49, 0.20, 0.34, and 0.64, respectively. Conversely, for *M. ruthenicus*, the average NST values were 0.37, 0.08, 0.38, and 0.94 for the CK, W, P, and WP treatments, respectively, indicating that the community assembly processes changed from deterministic-dominated to stochastic-dominated under the combined treatment. For *E. nutans*, the NST of the root endophytic bacterial community showed a significant negative correlation with soil TC and TN. For *M. ruthenicus*, the NST of the root endophytic bacterial community showed a significant positive correlation with soil TP (Table 2). We found no significance between the NST and soil properties for *K. humilis*.

### 3.3. Differences in Bacterial Community Composition among Plant Species

**Figure 5:**
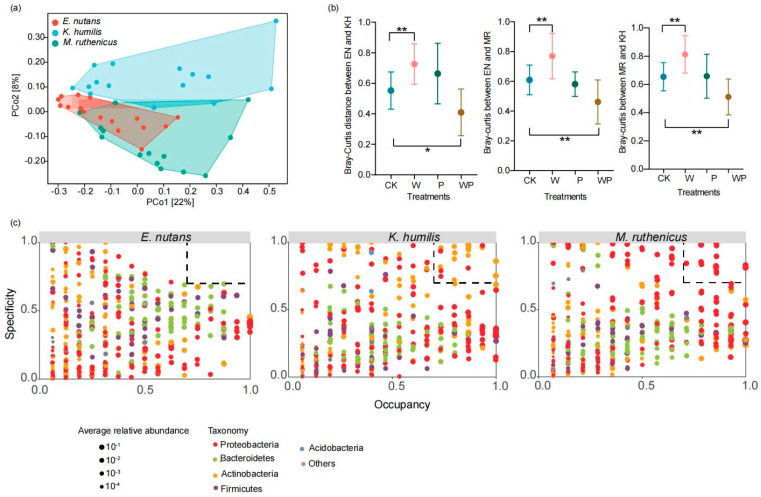
Differences in endophytic bacterial communities among the three plant species. (**a**) The principal coordinate analysis (PCoA) plot, (**b**) Bray–Curtis distance between two of the three plant species under different treatments, and (**c**) SPEC-OCCU diagrams of each plant species. EN, *E. nutans*; KH, *K. humilis*; MR, *M. ruthenicus*. CK, control; W, warming; P, precipitation increase; WP, a combination of warming and precipitation increase. * indicates significant difference at the 0.05 level, and ** indicates significant difference at the 0.01 level.

The PCoA plot showed a distinct segregation of the bacterial communities between *E. nutans* and *K. humilis* (Anosim, R = 0.12, *p* = 0.009), *E. nutans* and *M. ruthenicus* (Anosim, R = 0.19, *p* = 0.004), and *K. humilis* and *M. ruthenicus* (Anosim, R = 0.17, *p* = 0.002) (Figure 5a). The dissimilarity of the bacterial communities between two of the three plant species increased under the warming treatment. However, they decreased under the combined treatment (Figure 5b). The specificity and occupancy of the ASVs were calculated to identify the key bacterial ASVs in each plant species. As indicated by the SPEC-OCCU plots, the occupancy of most ASVs varied greatly for all three plant species (Figure 5c). Specialist ASVs were not detected in *E. nutans*. The specialist ASVs for *K. humilis* mainly belonged to the phyla Actinobacteria and Proteobacteria for *M. ruthenicus*.

### 3.4. Correlations between Endophytic Bacterial Community Composition and Soil Characteristics

**Figure 6:**
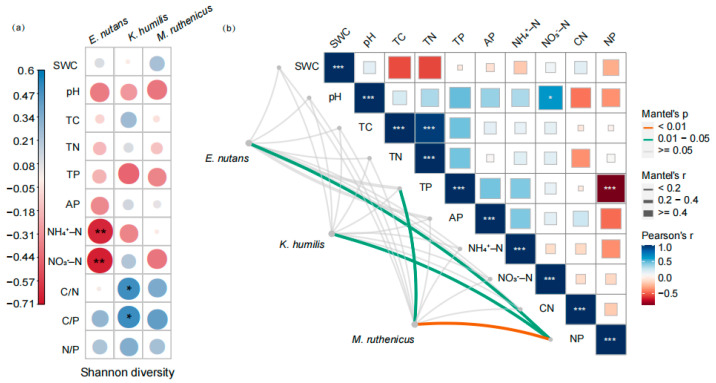
Correlations between Shannon diversity (**a**) and composition (**b**) of endophytic bacterial communities and soil properties. * indicates significant correlation at the 0.05 level, and ** indicates significant correlation at the 0.01 level. *** indicates significant correlation at the 0.001 level. SWC, soil water content; pH, soil pH; TC, soil total carbon content; TN, soil total nitrogen content; TP, soil total phosphorus content; AP, soil available phosphorus content; NH_4_^+^-N, soil ammonium nitrogen content; NO_3_^−^-N: soil nitrate nitrogen content; C/N, soil carbon to nitrogen ratio; C/P, soil carbon to phosphorus ratio; N/P, soil nitrogen to phosphorus ratio.

The bacterial Shannon diversity of *E. nutans* showed a significant negative correlation with soil ammonium nitrogen and nitrate nitrogen content, whereas that of *K. humilis* was positively correlated with the soil C/N and C/P ratios. No significant correlations were observed between the bacterial Shannon diversity index and the soil properties for *M. ruthenicus* (Figure 6a). According to the Mantel test, the root endophytic bacterial community compositions were significantly correlated with soil N/P ratio for *E. nutans*, *K. humilis*, and *M. ruthenicus* (Figure 6b).

The correlations between the soil properties and the relative abundances of the dominant bacterial families were analyzed further (Figure S1). The results indicated that specific bacterial families in different plant roots had different relationships with soil factors. The relative abundance of Burkholderiaceae was negatively correlated with the soil C/P and N/P ratios for *E. nutans* and *K. humilis* but not for *M. ruthenicus*. The relative abundance of Halomonadaceae was positively correlated with soil ammonium nitrogen content for *E. nutans* and positively correlated with soil total phosphorus content for *K. humilis*. However, it was negatively correlated with the soil C/N ratio for *M. ruthenicus*. The relative abundance of Aeromonadaceae was positively correlated with soil pH for all three plant species.

## 4. Discussion

Generally, we found that a 2 °C warming and a 20% precipitation increase had a negligible effect on root endophytic bacterial community composition. Other studies have also found inconspicuous changes in endophytic communities under fertilization or other anthropogenic interference [38,39,40]. These results indicated that plants can maintain the stability of their endophytic communities. The response of root endophytic communities to environmental changes is host-specific. In the present study, the changes in the alpha diversity and composition of the root endophytic communities of *E. nutans* were more significant under warmer and wetter climatic conditions than those of *K. humilis* and *M. ruthenicus*. *E. nutans* is a widespread Poaceae species in alpine grasslands, and it responded quickly to soil nutrients, especially soil available nitrogen concentration. Soil NH_4_^+^-N and NO_3_^−^-N contents increased significantly under the combined treatment, which might trigger a series of plant responses including regulation of the root microbiota to achieve the effective utilization of soil nutrients. The root endophytic bacterial communities of *K. humilis* and *M. ruthenicus* remained unchanged under climate changes, which indicated that their hosts provided a relatively stable internal environment and that the response of these two plants to climate change might be delayed. Endophytes play an important role in improving plant environmental adaptability, but the degree of their influence varies among plant species. The different sensitivities of endophytic communities to climate change reflect differences in the efficiency and capability of plants to manipulate endophytic communities. Plant species with higher regulation ability of their endophytic community might have a stronger competitive advantage in vegetation communities under future climate scenarios.

Soil nutrients are the main source of plant nutrients, and soil C, N, and P concentrations are crucial for determining plant growth rate and nutrient use efficiency [41]. The soil’s nutrient content is important for root endophytic communities. Wei et al. (2002) reported that the bacterial alpha diversity in *E. nutans* roots is regulated by soil carbon and nitrogen contents on large geographical scales [30]. A significant negative correlation between the bacterial alpha diversity of *E. nutans* roots and soil available nitrogen content was found in this study. The increased soil available nitrogen content under the combined warming and increased precipitation treatments might have contributed to the decreases in the root bacterial diversity. Notably, a reduction in endophytic diversity does not always negatively affect host plants. Allsup reported that a lower fungal richness conferred an enhancement in the cold tolerance of tree seedlings [42]. The soil C/N ratio reflects the soil nitrogen mineralization capacity, which affects plant nitrogen absorption. Legumes are less dependent on soil nitrogen availability because of their nitrogen-fixing rhizobia. No significant correlations were found between the root endophytic bacterial communities of *M. ruthenicus* and the soil nitrogen content or the soil C/N ratio. The soil C/P ratio is generally regarded as a sign of the organic phosphate mineralization ability in soil, where a higher C/P leads to phosphorus limitation during the decomposition of organic matter, which is not conducive to plant growth [43]. The Shannon diversity of the root endophytic bacterial community of *K. humilis* was positively correlated with the soil C/P ratio, implying that the endophytic bacterial diversity would increase in phosphorus-restricted soils. As a predictor of plant nutrient restriction, the soil N/P ratio could affect biological nitrogen fixation and plant nutrient absorption. The soil N/P ratio affects the N/P ratio in plants, which regulates plant growth. The soil N/P ratio was an important indicator of the root endophytic bacterial community composition in the present study. Specifically, the soil N/P ratio affected the relative abundance of Burkholderiaceae in the roots of *E. nutans* and the relative abundances of Pseudonocardiaceae and Micromonosporaceae in *K. humilis* roots. The composition of the endophytic bacterial community might be optimized by adjusting the soil nutrient content and soil stoichiometric ratio.

The relative abundance of Burkholderiaceae increased under the combined treatment of warming and increased precipitation for all three plant species. Many species and strains of Burkholderiaceae promote processes related to plant growth, including nitrogen fixation [44], phosphate solubilization [45], and IAA production [46]. The enrichment of Burkholderiaceae may be useful for alpine plants in adapting to warmer and humid climates. The relative abundance of Burkholderiaceae was affected by the soil C/P ratio, and higher soil phosphorus might contribute to the enrichment in Burkholderiaceae in plant roots. Bacteria of the Aeromonadaceae family have been reported to be very important plant-beneficial bacteria, which can increase plant stress resistance by producing the phytohormone auxin, ACC deaminase (1-aminocyclopropane-1-carboxylate), and decomposing extracellular hydrogen peroxide (H_2_O_2_) [47]. *K. humilis* has strong adaptability to the cold climate at high altitudes [48]. The significant enrichment of Aeromonadaceae in *K. humilis* roots under the combined treatment might reflect a state of duress in a warmer and wetter climate, which is not conducive to the species’ competition in the plant community.

Many previous studies have shown that endophytic community compositions differ among plant species, which are determined largely by the host plant’s phylogenetic relationships [12,26,49]. In addition, the degree of difference could be regulated by environmental factors such as climate and soil nutrient content [30]. Significant differences in the root endophytic bacterial communities between pairs of the three plant species were measured in this study. The degree of difference was magnified under the individual warming treatment and narrowed under the combined treatment. Jiang et al. (2021) also found an enhanced dissimilarity in the root-associated fungal community composition among 14 plant species under climate warming on the Qinghai-Tibetan Plateau [50]. Differences in the composition of endophyte communities further reflect the functional and adaptive separation of co-occurring plant species. The climate of the Qinghai-Tibet Plateau is cold and dry, and warming might exacerbate the drought of the topsoil and reduce the soil’s nutrient availability. In the case of nutrient reduction, the differentiation of endophytic community compositions among coexisting plant species may be a strategy to reduce interspecific competition. Endospheric dissimilarity among plant species was positively correlated with plant–soil feedback, and focal plant species performed better under soil conditions planted with highly dissimilar root microbial communities [49]. The results demonstrated that the similarity of the endophytic communities among the three plant species was altered under simulated climate change conditions, which might have changed the results of the plant–soil feedback. Plant species tended to share more dissimilar root endophytic communities under individual warming treatments, which might have led to positive plant–soil feedback and promoted interspecies coexistence. However, the results showed the opposite under the combined treatment, which might have intensified interspecific competition. These results provide important reference values for exploring vegetation community succession under climate change conditions.

Community assembly processes are critical for understanding changes in community diversity and composition. Previous studies have reported that the endophytic community assembly patterns of quinoa [51] and desert shrubs [52] are generally dominated by stochastic processes. Meanwhile, deterministic processes dominated the endosphere microbiomes of mangroves [53] and grapevine [54]. We found that the effects of climate change on endophytic community assembly processes were host-specific. Community assembly processes shifted from stochastic to deterministic processes under the combined treatment for *E. nutans*. In contrast, the opposite shift was observed for *M. ruthenicus*, suggesting that the changes in the endophytic bacterial communities of *E. nutans* were more susceptible to environmental factors under warmer and wetter conditions. Some stochastic processes, such as ecological drift and diversification, were more frequent for *M. ruthenicus*. The microbial groups that are conducive to plant growth maintain a stable genetic symbiotic relationship with the host and are selectively enriched by the root system under specific conditions. Therefore, deterministic processes are often related to the active selection and adaption of host plants. Microbial communities processed by stochastic processes may invade plant roots opportunistically and do not possess beneficial effects on plants [52]. We surmised that the observed higher proportion of deterministic processes for *E. nutans* reflected its stronger ability to actively regulate endophytic bacteria than *M. ruthenicus* and *K. humilis*. Studies have reported that endophytic community assembly processes were affected by season [55], agricultural management [56], and the growth stage of plants [57]. We found that soil nutrients affected the assembly processes of the root endophytic communities. Furthermore, the NST of *E. nutans* was affected by soil TC and TN, and the NST of *M. ruthenicus* was affected by soil TP. These soil nutrients affect endophytic community assemblies by influencing the physiological characteristics of host plants.

## 5. Conclusions

In the present study, we investigated the effects of warming and precipitation increases on bacterial community diversity, composition, and assembly processes in the root endosphere of three coexisting plant species in an alpine grassland. The specialist species and community composition of the root endophytes differed among the three plant species. Furthermore, we found that the root endophytic community structure, assembly processes, and responses to climate change were host-specific. Individual warming and precipitation increases had no significant effects on the diversity or composition of the root endophytic bacterial communities. The combination of warming and precipitation increase caused a noticeable drop in the Shannon index of the bacterial community, for which that the grass species decreased the most. Additionally, we observed a remarkable shift in the relative importance of stochastic and deterministic processes under the combined treatment. Soil available nitrogen content and stoichiometric ratios are important regulators of root endophytic bacterial community structure. The present study enhances our understanding of root endophytic communities under climate change in the Qinghai-Tibet Plateau.

## Figures and Tables

**Table 1:** Results of PERMANOVA analysis revealing differences in bacterial communities between treatments for three plant species.

Plant Species	Treatment			*F*	*P*
*Elymus nutans*	CK	vs.	W	1.40	0.11
	CK	vs.	P	1.40	0.14
	CK	vs.	WP	2.75	0.03
	W	vs.	P	0.73	0.94
	W	vs.	WP	2.89	0.02
	P	vs.	WP	2.27	0.02
*Kobresia humilis*	CK	vs.	W	1.82	0.11
	CK	vs.	P	1.57	0.14
	CK	vs.	WP	1.48	0.18
	W	vs.	P	0.81	0.72
	W	vs.	WP	2.34	0.05
	P	vs.	WP	1.84	0.08
*Melissilus ruthenicus*	CK	vs.	W	1.54	0.09
	CK	vs.	P	1.07	0.35
	CK	vs.	WP	1.64	0.12
	W	vs.	P	1.27	0.19
	W	vs.	WP	1.50	0.21
	P	vs.	WP	1.38	0.08

Notes: CK, control; W, warming; P, precipitation increase; WP, a combination of warming and precipitation increase.

**Table 2:** Correlations between normalized stochasticity ratio of root endophytic bacterial community and soil properties.

	Normalized Stochasticity Ratio		
	*E. nutans*	*K. humilis*	*M. ruthenicus*
SWC/%	0.80	0.40	0.20
pH	−0.21	0.63	0.95
TC (g/kg)	−1.00 **	0.20	0.40
TN (g/kg)	−1.00 **	0.20	0.40
TP(g/kg)	−0.40	0.80	1.00 **
AP (mg/kg)	−0.40	0.00	0.60
NH_4_^+^-N (mg/kg)	−0.80	0.40	0.80
NO_3_^−^-N (mg/kg)	−0.40	0.00	0.60
C/N	0.00	−0.40	−0.80
N/P	0.00	−0.40	−0.80

Notes: The numbers in the table refer to the Spearman correlation coefficient, and ** indicates significant correlation at the 0.01 level. SWC, soil water content; pH, soil pH; TC, soil total carbon content; TN, soil total nitrogen content; TP, soil total phosphorus content; AP, soil available phosphorus content; NH_4_^+^-N, soil ammonium nitrogen content; NO_3_^−^-N: soil nitrate nitrogen content; C/N, soil carbon to nitrogen ratio; N/P, soil nitrogen to phosphorus ratio.

## Data Availability

The MiSeq data will be openly available from the NCBI repository upon publication.

## Supplementary Materials

The following supporting information can be downloaded at: https://www.mdpi.com/article/10.3390/microorganisms12091780/s1, Figure S1: Spearman correlations between soil properties and relative abundances of dominant bacterial families for *Elymus nutans* (A), *Kobresia humilis* (B), *Melissilus ruthenicus* (C).
