# Supplementary material for: Shifts in Structure and Assembly Processes of Root Endophytic Community Caused by Climate Warming and Precipitation Increase in Alpine Grassland
Source: Microorganisms. 2024 Aug 28;12(9):1780. doi: 10.3390/microorganisms12091780 (PMC11434594; doi:10.3390/microorganisms12091780)
Supplement: Supplementary file 1 [file microorganisms-12-01780-s001.zip › microorganisms-3143177-supplementary.pdf]

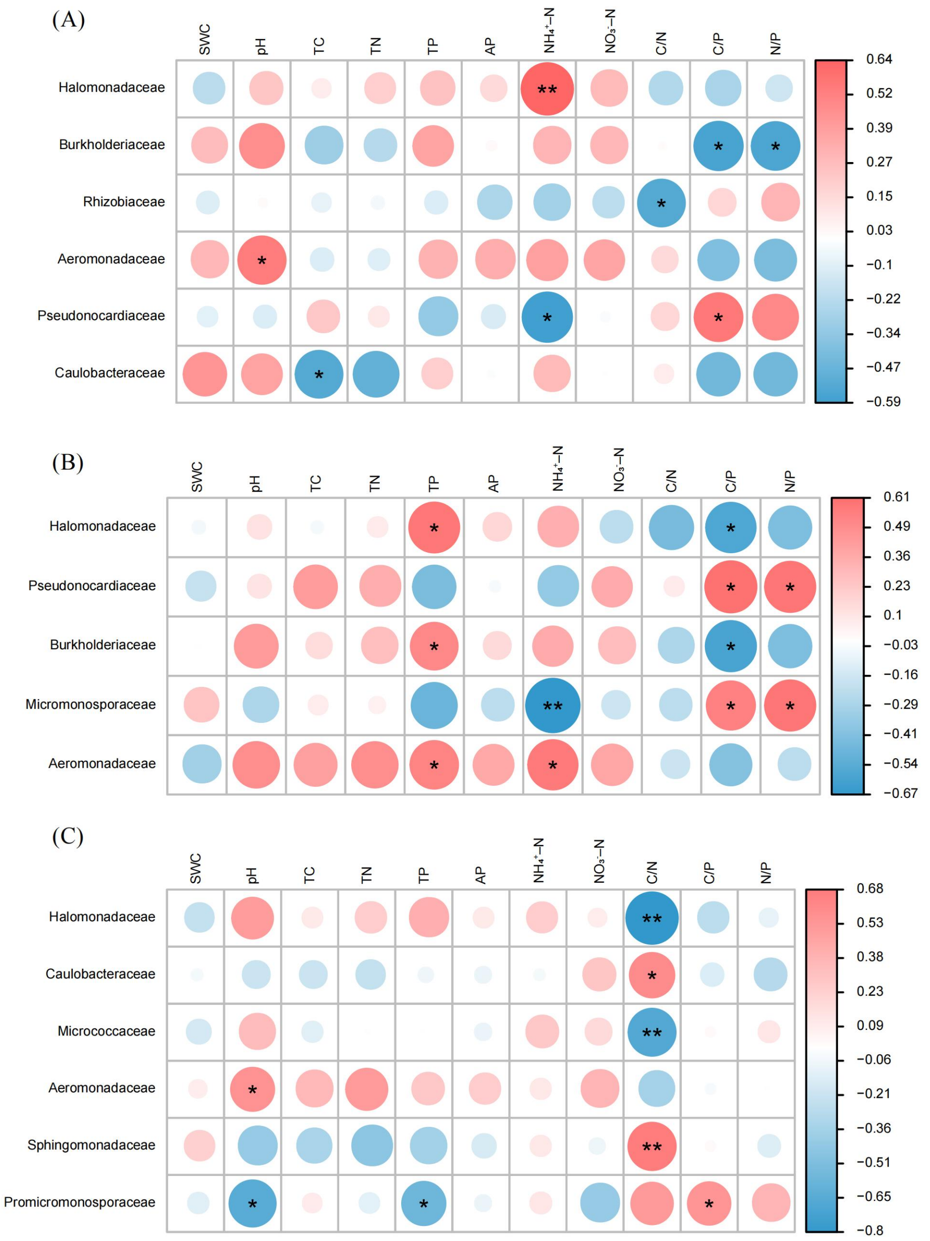

Figure S1. Spearman correlations between soil properties and the relative abundance of dominant bacterial families for *Elymus nutans* (A), *Kobresia humilis* (B), *Melissilus ruthenicus* (C). * indicates significant correlations at the 0.05 and ** at the 0.01 levels. The number represents the correlation coefficient, and the positive number represents the positive correlation, and the negative number represents the negative correlation. The darker the color, the stronger the correlation. SWC, soil water content; pH, soil pH; TC, soil total carbon content; TN, soil total nitrogen content; TP, soil total phosphorus content; AP, soil available phosphorus content; NH_4^+-N, soil ammonium nitrogen content; $NO3^-$-N: soil nitrate nitrogen content; C/N, Soil carbon to nitrogen ratio; N/P, soil nitrogen to phosphorus ratio; C/P, Soil carbon to phosphorus ratio.
